# A Resumable Fluorescent Probe BHN-Fe_3_O_4_@SiO_2_ Hybrid Nanostructure for Fe^3+^ and its Application in Bioimaging

**DOI:** 10.1186/s11671-017-2392-2

**Published:** 2017-12-19

**Authors:** Xi Zhou, Yujiao Wang, Qi Peng, Weisheng Liu

**Affiliations:** 1 Key Laboratory of Nonferrous Metals Chemistry and Resources Utilization of Gansu Province, State Key Laboratory of Applied Organic Chemistry, College of Chemistry and Chemical Engineering, Lanzhou University, Lanzhou, 730000 China; 2 Department of Pathogenic Biology, School of Basic Medical Sciences, Lanzhou University, Lanzhou, 730000 China

**Keywords:** Fluorescent probe, Resumable, Hybrid nanostructure, Fe^3+^, Bioimaging

## Abstract

A multifunctional fluorescent probe BHN-Fe_3_O_4_@SiO_2_ nanostructure for Fe^3+^ was designed and developed. It has a good selective response to Fe^3+^ with fluorescence quenching and can be recycled using an external magnetic field. With adding EDTA (2.5 × 10^−5^ M) to the consequent product Fe^3+^-BHN-Fe_3_O_4_@SiO_2_, Fe^3+^ can be removed from the complex, and its fluorescence probing ability recovers, which means that this constituted on-off type fluorescence probe could be reversed and reused. At the same time, the probe has been successfully applied for quantitatively detecting Fe^3+^ in a linear mode with a low limit of detection 1.25 × 10^−8^ M. Furthermore, the BHN-Fe_3_O_4_@SiO_2_ nanostructure probe is successfully used to detect Fe^3+^ in living HeLa cells, which shows its great potential in bioimaging detection.

**Electronic supplementary material:**

The online version of this article (10.1186/s11671-017-2392-2) contains supplementary material, which is available to authorized users.

## Background

The development of new methods to detect all kinds of small molecules and ions has become an important task for scientific researchers. As one of the indispensable important metal ions in metabolic processes, Fe^3+^ plays an essential and crucial role in a variety of biological processes such as brain function and pathology, gene transcription, immune function, and mammalian reproduction [1–9]. The medical investigations indicate that the metabolic or biological processes are normal for the proper functioning of all living cells only when the Fe^3+^ concentration is in a suitable range. When Fe^3+^ concentration in a living body deviates from its suitable range, some diseases or serious disorders can be induced in the metabolic or biological processes [10–12]. Even though a variety of detection methods has been developed to detect Fe^3+^ [13–15], fluorescent technique is the more effective and powerful method, because of their operational simplicity, high sensitivity and selectivity, and low detection limit [16–20].

In these molecule-based fluorescent probes, some problems relative to the safety, the recyclability, and the reusability have not been solved. For example, as pointed out in reference [21], the employed small molecules are toxic. These deficiencies exhibited in the molecule-based fluorescent probes completely limit the probes entering into a practical application. To conquer the challenge of safety in the above fluorescent probes for Fe^3+^, another technical approach is proposed by using inorganic supports incorporated with small molecular fluorescent probes. In such new approach, it is known that the inorganic materials such as magnetic nanoparticles, metal nanoparticles, nanotubes, and mesoporous silica can be used in the design of the fluorescent probes [22–24]. Among all these inorganic materials, magnetic silica core-shell Fe_3_O_4_@SiO_2_ nanoparticles have advantages of their low toxicity, high biocompatibility, simply separation via external magnetic field, and large surface area that can be grafted by fluorescent probes over other materials in the molecule or ion recognition and separation areas [25–27]. Hence, this new approach provides us a possible way to realize the application for detecting Fe^3+^, especially in the safety with low toxicity and high biocompatibility.

In this work, a kind of multifunctional magnetic BHN-Fe_3_O_4_@SiO_2_ nanostructure fluorescent sensor for Fe^3+^ was designed and synthesized. It has a good sensitive and selective response to Fe^3+^ with remarkably fluorescence quenching in CH_3_CN/H_2_O (1:1, *v*/*v*) at room temperature. By applying an external magnetic field, the probe can be separated from the solution. When adding EDTA to the system, Fe^3+^ can be removed from the complex with fluorescence intensity recovery. Furthermore, the confocal fluorescence imaging using HeLa cells showed that the probe could be applied to detect Fe^3+^ in living cells. Hence, the obtained BHN-Fe_3_O_4_@SiO_2_ exhibits excellent selectivity, water solubility, reversibility, and recyclability, which benefits to the detection of Fe^3+^.

## Methods/Experimental

### Synthesis of Fe_3_O_4_@SiO_2_ Nanoparticles

Fe_3_O_4_ magnetite nanoparticles were synthesized according to reference [28]. They were further coated with a thin silica layer by means of a modified Stöber method [29] to obtain stable Fe_3_O_4_@SiO_2_. Tetraethyl orthosilicate (TEOS) was hydrolyzed with magnetite nanoparticles as seeds in ethanol/water mixture. The resulting Fe_3_O_4_@SiO_2_ nanoparticles with an average diameter of 50–60 nm were used as the carriers of fluorescent sensor nanoparticles.

### Synthesis of BHN-Fe_3_O_4_@SiO_2_ Nanostructure

*N*-butyl-4-bis(2-hydroxyethyl) amino-1,8-naphthalimide (BHN) is synthesized according to the method reported before [30, 31]. The first intermediate was synthesized by the reaction between 4-bromo-1,8-naphthalic anhydride and *n*-butylamine. Then, the intermediate reacted with diethanolamine to afford BHN. ESI-MS: m/z 357.3 (M + H^+^). ^1^H NMR (CDCl_3_, 400 MHz): *δ* (ppm): 0.95 (t, 3H, *J* = 8.0 Hz); 1.41(m, 2H); 1.66 (m, 2H); 2.69 (m, 2H); 3.60 (t, 4H, *J* = 5.0 Hz); 3.86(t, 4H, *J* = 5.0 Hz); 4.08 (t, 2H, *J* = 8.0 Hz); 7.33 (d, 1H, *J* = 8.0 Hz); 7.58 (t, 1H, *J* = 8.0 Hz); 8.38(d, 1 H, *J* = 8.0 Hz); 8.41 (dd, 1H, *J* = 8.0 Hz); 8.84 (dd, 1H, *J* = 8.0 Hz).

BHN (356 mg, 1 mmol) and 3-isocyanatopropyl-triethoxysilane (IPTES, 494 mg, 2 mmol) were mixed in anhydrous THF (15 mL) at room temperature. Then the solution was refluxed for 48 h under N_2_. After that, the solvent was evaporated, and the crude product was further purified by flash column chromatography (silica gel, petroleum ether/CH_2_Cl_2_/methanol 50/50/1) to afford 255 mg (30%) of BHN-IPTES as a yellow powder. ESI-MS: m/z 851.5(M + H^+^). ^1^H NMR: (400 MHz, CDCl_3_): *δ* (ppm) 0.60 (t, 4H, *J* = 8.0 Hz); 0.98 (t, 3H, *J* = 8.0 Hz); 1.21 (m, 18H); 1.45 (m, 2H); 1.58 (m, 4H); 1.70 (m, 2H); 3.13 (m, 4H); 3.73 (t, 2H, *J* = 5.0 Hz); 3.82 (m, 12H); 4.16 (m, 4H); 4.24 (m, 4H); 4.94 (m, 2H); 7.38 (d, 1H, *J* = 8.0 Hz); 7.70 (t, 1H, *J* = 8.0 Hz); 8.45 (d, 1H, J = 8.0 Hz); 8.50 (dd, 1H, *J* = 8.0 Hz); 8.58 (dd, 1H, *J* = 8.0 Hz).

One hundred milligrams of dried Fe_3_O_4_@SiO_2_ nanoparticles and 300 mg (0.35 mmol) of BHN-IPTES were suspended in anhydrous toluene (15 mL). The solution was refluxed for 12 h at 110 °C under N_2_ to obtain BHN-Fe_3_O_4_@SiO_2_. The nanoparticles were collected by centrifugation (10,000 rpm) and repeatedly washed with anhydrous ethanol thoroughly. By monitoring the fluorescence of the upper liquid, unreacted organic molecules could be removed completely. Then, the BHN-Fe_3_O_4_@SiO_2_ nanostructure was finally dried under vacuum overnight.

## Results and Discussion

### Design of BHN-Fe_3_O_4_@SiO_2_

Fe_3_O_4_@SiO_2_ nanoparticle is a promising candidate to construct safe, recyclable, and reusable Fe^3+^ fluorescent sensor due to its low toxicity, high biocompatibility, and convenient recyclability via external magnetic field. Compared with other fluorophores, 1,8-naphthalimide has a large Stokes’ shift, long emission wavelength, and convenience to modify with different side-chain and high quantum yield. So, with the introduction of proper side-chain, it can be grafted on the Fe_3_O_4_@SiO_2_ nanoparticle to obtain a safe, recyclable, and reusable Fe^3+^ fluorescent sensor with remarkable fluorescence response.

**Fig. 1:**
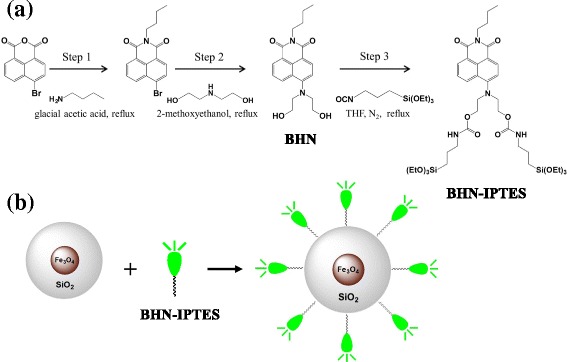
**a** Synthesis of BHN. **b** Synthesis of BHN-Fe_3_O_4_@SiO_2_

As is well known, Fe^3+^ can be easily coordinated with O and N atom, so we modified 1,8-naphthalimide with diethanolamine to make the 1,8-naphthalimide possess the ability to detect Fe^3+^ as shown in Fig. 1a. In the diethanolamine, hydroxyethyl and ester-amide moieties were served as a receptor unit. Finally, the modified 1,8-naphthalimide was grafted on the Fe_3_O_4_@SiO_2_ via hydrolysis-condensation reaction between Si (OEt)_3_ and hydroxyl in the surface of Fe_3_O_4_@SiO_2_ as shown in Fig. 1b.

### Structure of BHN-Fe_3_O_4_@SiO_2_

**Fig. 2:**
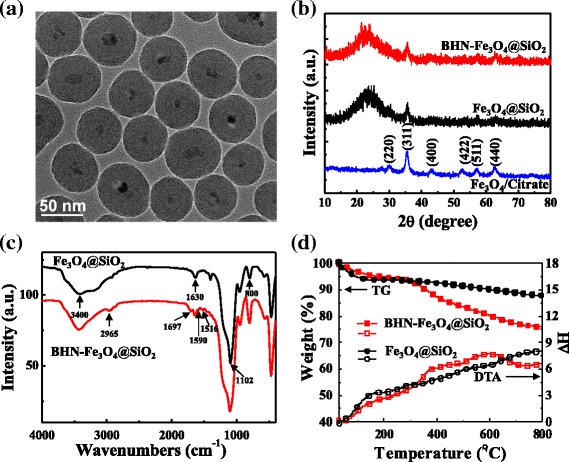
**a** TEM image of BHN-Fe_3_O_4_@SiO_2_ (the scale bar is 50 nm.). **b** XRD patterns of Fe_3_O_4_/Citrate, Fe_3_O_4_@SiO_2_, and BHN-Fe_3_O_4_@SiO_2_. **c** FT-IR spectra of Fe_3_O_4_@SiO_2_ and BHN-Fe_3_O_4_@SiO_2_. **d** TG and DTA curves of Fe_3_O_4_@SiO_2_ and BHN-Fe_3_O_4_@SiO_2_

From the TEM image as shown in Fig. 2a, the typical core/shell structure of BHN-Fe_3_O_4_@SiO_2_ is clearly displayed. Although the bare magnetic core is easy to aggregate in liquid, the silica shell on the surface of magnetic nanoparticles would prevent aggregation and improve the dispersibility. The iron oxide nanoparticles have been entrapped in the silica shell successfully and dispersed well. It can also be seen that the overall diameters of core/shell structures are in a narrow distribution of 50 to 60 nm with iron oxide core of 10 nm, which is lower than its superparamagnetic critical size and suitable for using as fluorescent probe’s carrier nanoparticle.

Figure 2b shows the XRD powder diffraction patterns of Fe_3_O_4_, Fe_3_O_4_@SiO_2_, and BHN-Fe_3_O_4_@SiO_2_. The six characteristic diffraction peaks of bare Fe_3_O_4_ can be indexed to 220, 311, 400, 422, 511, and 440 reflections of the magnetite. However, the XRD peaks attributed to Fe_3_O_4_ have low intensities in Fe_3_O_4_@SiO_2_ and BHN-Fe_3_O_4_@SiO_2_, which implies that the Fe_3_O_4_ nanoparticles are coated with amorphous silica shell. The silica shell may decrease the relative content of Fe_3_O_4_ cores and then affect the peak intensities. Also, the broad XRD package is found at a low diffraction angle of 20° to 30° in Fe_3_O_4_@SiO_2_ and BHN-Fe_3_O_4_@SiO_2_, which corresponds to the amorphous-state SiO_2_ shells surrounding the Fe_3_O_4_ nanoparticles.

To study the modification condition of BHN-IPTES on the surface of the Fe_3_O_4_@SiO_2_ nanoparticles, its Fourier transform infrared (FT-IR) spectrum is measured. As shown in Fig. 2c, both two curves exhibited the typical vibration band of −OH stretching on silanol at 3400 to 3500 cm^−1^ and 1000 to 1200 cm^−1^ [32]. It indicates that not all the silanol on Fe_3_O_4_@SiO_2_ nanoparticles have been covalently modified. The band at 1630 cm^−1^ represents the bending mode of −OH vibrations [33]. The bands centered at 1109 (*νas*) and 800 cm^−1^ can be attributed to the siloxane (-Si-O-Si-) [34]. The above peaks indicate the existence of silica shell. The additional peaks at 2965 and 2934 cm^−1^ were found in BHN-Fe_3_O_4_@SiO_2_, corresponding to the −CH vibration of aliphatic and aromatic groups [32, 35]. The band at 1697, 1590, and 1516 cm^−1^ of BHN-Fe_3_O_4_@SiO_2_ comes from the bending vibrations of −CH_3_ from the BHN part [36]. These results demonstrate the presence of the organic molecule in the magnetic material BHN-Fe_3_O_4_@SiO_2_.

The superparamagnetic property of the magnetic nanoparticles plays a vital role for its biological application. Additional file 1: Figure S1 shows the magnetization curve of BHN-Fe_3_O_4_@SiO_2_ which was measured by a vibrating sample magnetometer in the range from − 15,000 to 15,000 Oe at 300 K. The result was consistent with the conclusion that the diameter of magnetic Fe_3_O_4_ nanoparticles less than 30 nm is usually superparamagnetic at room temperature [37]. The saturation magnetization value for synthesized BHN-Fe_3_O_4_@SiO_2_ is about 4.02 emu/g. More importantly, from the hysteresis loop of BHN-Fe_3_O_4_@SiO_2_ nanostructure, it can be found that it exhibited superparamagnetic properties, and no coercive force was observed in the hysteresis loop. This phenomenon was due to the fact that the magnetite core has a small diameter around 10 nm. At the same time, the silica shell prevents the aggregation of magnetite core. So, the BHN-Fe_3_O_4_@SiO_2_ nanostructure can further show good dispersibility.

## Fluorescence Response of BHN-Fe_3_O_4_@SiO_2_

**Fig. 3:**
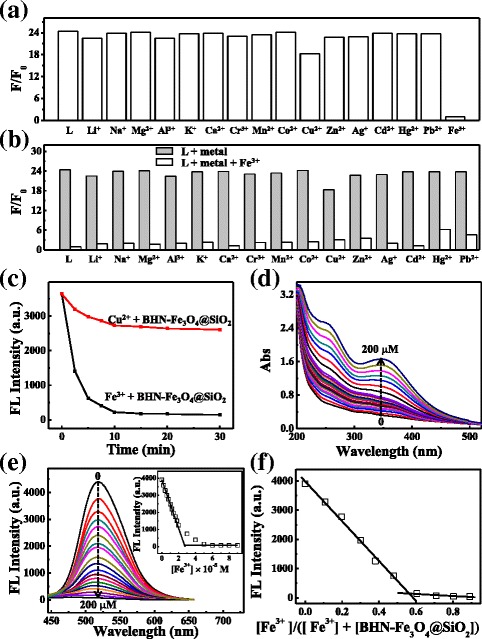
**a** Fluorescence responses of BHN-Fe_3_O_4_@SiO_2_ with various cations. Excitation wavelength was 415 nm. Spectra were recorded every 2 min after adding metal ions. **b** Competition of Fe^3+^-BHN-Fe_3_O_4_@SiO_2_ towards cations. Fluorescent emission change of BHN-Fe_3_O_4_@SiO_2_ (0.2 g/L) upon addition of metal ions (each metal ion is 5 × 10^−5^ M) in CH_3_CN/H_2_O 1:1 (HEPES buffer pH 7.36) at room temperature. **c** Time responses of BHN-Fe_3_O_4_@SiO_2_ with Fe^3+^ and Cu^2+^. **d** UV-Vis titrations of BHN-Fe_3_O_4_@SiO_2_ (0.2 g/L) with Fe^3+^. **e** Fluorescence titration of BHN-Fe_3_O_4_@SiO_2_ (0.2 g/L) with Fe^3+^. Inset: the fluorescence intensities at 518 nm at various concentrations of Fe^3+^. **f** Job’s plot of BHN-Fe_3_O_4_@SiO_2_ with Fe^3+^

To verify the fluorescence response of BHN-Fe_3_O_4_@SiO_2_ for various metal ions, the fluorescence measurements were carried out in CH_3_CN/H_2_O 1:1 (*v*/*v*) solution at pH 7.36 in HEPES buffer. The concentration of BHN-Fe_3_O_4_@SiO_2_ is 0.2 g/L (corresponding to the free organic molecule was about 3.34 × 10^−5^ M, according to the TGA, see Fig. 2d), and the various metal ions Ag^+^, Al^3+^, Ca^2+^, Cd^2+^, Co^2+^, Cr^3+^, Cu^2+^, Hg^2+^, K^+^, Li^+^, Mg^2+^, Mn^2+^, Na^+^, Pb^2+^, Zn^2**+**^, and Fe^3+^ (all as their perchlorates salts) were 5.0 × 10^−5^ M. As shown in Fig. 3a, a significant fluorescence quenching was observed when adding Fe^3+^, but no significant decrease of fluorescent intensity in the same conditions was detected if adding other metal ions except Cu^2+^. Cu^2+^ would cause slight fluorescence quenching and response in 20 min. However, at the same detecting conditions, Fe^3+^ causes a response in 2 min and quench obviously in 5 min (Fig. 3c). The absorption spectra of BHN-Fe_3_O_4_@SiO_2_ (0.2 g/L) in the presence of various concentrations of Fe^3+^ (0 to 200 μM) were investigated, as shown in Fig. 3d. When Fe^3+^ was added gradually, the absorbance of BHN-Fe_3_O_4_@SiO_2_ at 250 and 350 nm gradually increases, which indicated that BHN-Fe_3_O_4_@SiO_2_ nanostructure coordinated with Fe^3+^ gradually.

Then, a fluorescence titration with Fe(ClO_4_)_3_ in CH_3_CN/H_2_O 1:1 (*v*/*v)* was applied to understand the combination of BHN-Fe_3_O_4_@SiO_2_ towards Fe^3+^ ions. As illustrated in Fig. 3e, the fluorescence emission of BHN-Fe_3_O_4_@SiO_2_ (0.2 g/L) decreases gradually when various concentrations (0 to 100 μM) of Fe^3+^ were added in CH_3_CN/H_2_O 1:1 (*v*/*v*) HEPES buffer, which indicates that BHN-Fe_3_O_4_@SiO_2_ nanostructure coordinated with Fe^3+^ to form the complex quantitatively. Fluorescence titration experiment suggests that the association constant log*β* for Fe^3+^ binding to BHN-Fe_3_O_4_@SiO_2_ is calculated to be 8.23. A linear increasing of fluorescence from the BHN-Fe_3_O_4_@SiO_2_ nanostructure was observed upon the addition of Fe^3+^ between 0 and 20 μM, and the limit of detection of BHN-Fe_3_O_4_@SiO_2_ to Fe^3+^ was found by 1.25 × 10^−8^ M under the fluorimetric assay. The fluorescence titration and Job plot results suggested a 1:1 binding ration for Fe^3+^ with BHN-Fe_3_O_4_@SiO_2_ (Fig. 3f). The results of cation competitive experiments are depicted in Fig. 3b, and it could be found that the selectivity and sensitivity of BHN-Fe_3_O_4_@SiO_2_ to Fe^3+^ are not influenced by other metal ions.

**Fig. 4:**
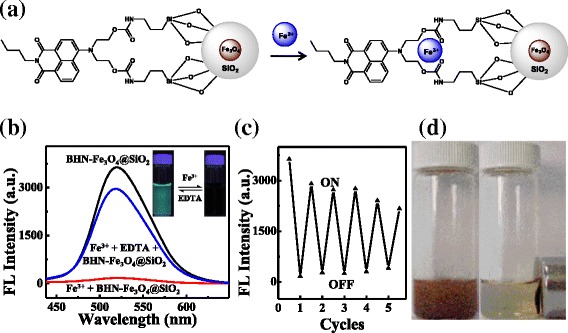
**a** Schematic show of BHN-Fe_3_O_4_@SiO_2_ with Fe^3+^. **b** Reversibility of BHN-Fe_3_O_4_@SiO_2_ towards Fe^3+^. Inset: the photograph of BHN-Fe_3_O_4_@SiO_2_ with Fe^3+^ by treatment of EDTA (2.5 × 10^−5^ M) under 415-nm UV light. **c** Plot of the fluorescence of BHN-Fe_3_O_4_@SiO_2_ (0.2 g/L) with alternate adding of 2.5 × 10^−5^ M Fe^3+^ (“off”) and EDTA (“on”). **d** BHN-Fe_3_O_4_@SiO_2_ (0.2 g/L) was dispersed to an external magnet in CH_3_CN/H_2_O 1:1 (HEPES buffer pH 7.36)

Here, the remarkable decrease of fluorescence intensity can be explained as follow: The fluorescence intensity of BHN-Fe_3_O_4_@SiO_2_, which is excited at a 415 nm lamp, exhibits the high fluorescence at 518 nm due to the 1,8-naphthalimide which has a big conjugated system. In addition, electron donating group in the structure influences the fluorescent of system at the same time. When stably chelated with Fe^3+^ by the O atom and N atom on the four-position of 1,8-naphthalimide, the electron or energy transfer between the metal cation and the fluorophore produce an electronic absorption effect, so as to make the fluorescence quenching [38] (Fig. 4a).

The fluorescence quenching by adding Fe^3+^ to the solution of BHN-Fe_3_O_4_@SiO_2_ was fully reversible. When adding EDTA (2.5 × 10^−5^ M) to the Fe^3+^-BHN-Fe_3_O_4_@SiO_2_ system, the fluorescence intensity was almost restored to the original level of BHN-Fe_3_O_4_@SiO_2_ (Fig. 4b). Further, reusability was evaluated by repeatedly adding Fe^3+^-EDTA cycles into the system, with the change of BHN-Fe_3_O_4_@SiO_2_ fluorescence intensity being recorded after each step, and the corresponding data are shown in Fig. 4c. It is clear that the BHN-Fe_3_O_4_@SiO_2_ exhibits excellent reusability because only the rare loss in BHN-Fe_3_O_4_@SiO_2_ sensitivity towards Fe^3+^ was observed after five repeated Fe^3+^-EDTA cycles. As a result of its magnetic property, BHN-Fe_3_O_4_@SiO_2_ had a reversal magnetic responsibility. As shown in Fig. 4d, it could be easily separated from the dispersion (0.2 g/L) after 10 min by placing a magnet closed to the dispersion, then redispersed by mild agitation when the magnet was removed. This magnetic separation capability and the recognition property of BHN-Fe_3_O_4_@SiO_2_ nanostructure provide a simple and efficient route to separate Fe^3+^ rather than through filtration approach. More important is that the reversal magnetic responsibility of BHN-Fe_3_O_4_@SiO_2_ nanostructure would be a key factor when evaluating their recyclability [39]. Combined with its magnetic property, it is demonstrated that BHN-Fe_3_O_4_@SiO_2_ was considerably applicable in the biological system as an efficient inorganic-organic hybrid sensor for Fe^3+^.

**Fig. 5:**
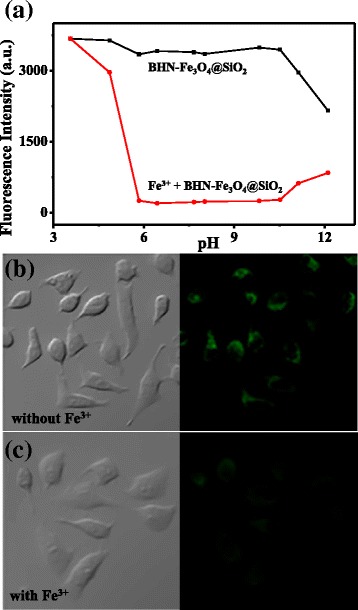
**a** Fluorescence intensities of BHN-Fe_3_O_4_@SiO_2_ and Fe^3+^-BHN-Fe_3_O_4_@SiO_2_ at various pH values at room temperature. CH_3_CN/H_2_O 1:1, *λ*_ex_ = 415 nm. **b** Bright field image and fluorescence image of the HeLa cells with BHN-Fe_3_O_4_@SiO_2_. (**c**) Bright field image and fluorescence image of the HeLa cells with BHN-Fe_3_O_4_@SiO_2_ and Fe^3+^

For the biological application, it is critically important that the sensor should be suitable for measuring specific metal ion in the physiological pH range. As shown in Fig. 5a, the fluorescence intensities of BHN-Fe_3_O_4_@SiO_2_ with/without Fe^3+^ at various pH values were investigated. The fluorescence intensity of BHN-Fe_3_O_4_@SiO_2_ slightly decreases when adding Fe^3+^ under acidic conditions, since protonation of N atom on the four-position of 1,8-naphthalimide leads to a weak coordination ability of Fe^3+^. Then, a dramatic fluorescence change for Fe^3+^-BHN-Fe_3_O_4_@SiO_2_ system was found when pH was at neutral pH and under weakly alkaline conditions. Here, BHN-Fe_3_O_4_@SiO_2_ exhibits excellent Fe^3+^ sensing abilities when the pH is in the range of 5.84 to 10.52, which indicates that BHN-Fe_3_O_4_@SiO_2_ is an expecting probe to be applied in those complicated environments or biological systems.

To further demonstrate the ability of BHN-Fe_3_O_4_@SiO_2_ to detect Fe^3+^ in living systems, we carried on an experiment in live HeLa cells. First of all, we investigated the cell viability of BHN-Fe_3_O_4_@SiO_2_ and Fe^3+^-BHN-Fe_3_O_4_@SiO_2_ using the MTT assay. HeLa cells were incubated with BHN-Fe_3_O_4_@SiO_2_ in RPMI-1640 for 0.5 h at 37 °C, and then Fe(ClO_4_)_3_ was added for incubation for 0.5 h. Then, the confocal fluorescence images of the HeLa cells were observed, and it shows excellent staining capacity when the concentration of the sensor and Fe(ClO_4_)_3_ is up to 0.2 g/L and 5 × 10^−5^ M. Then, we conducted fluorescence microscopy experiment to investigate its higher gradation of application in complex biological systems. As shown in Fig. 5b, HeLa cells were grown on 12 orifice plate at 37 °C and in 5% CO_2_ atmosphere for 24 h, then treated with BHN-Fe_3_O_4_@SiO_2_ (0.2 g/L) and incubated for 0.5 h, and the cells showed strong green fluorescence. Then, the cells were treated with 5 × 10^−5^ M Fe(ClO_4_)_3_. After 0.5 h, we did observe the fluorescent remarkably decreased (Fig. 5c). Thus, we can draw a conclusion that BHN-Fe_3_O_4_@SiO_2_ can be used to image Fe^3+^ in living cells.

## Conclusion

In summary, a novel multifunctional fluorescent probe BHN-Fe_3_O_4_@SiO_2_ nanostructure for Fe^3+^ was successfully designed and synthesized. The probe BHN-Fe_3_O_4_@SiO_2_ can selectively respond to Fe^3+^ with fluorescence quenching and efficient separation of Fe^3+^ with external magnetic field. The constituted on-off type fluorescence monitoring system indicates that the probe could be reversed back and reused. At the same time, the probe has been successfully applied to quantitatively detect Fe^3+^ with low detection limits. Furthermore, the BHN-Fe_3_O_4_@SiO_2_ nanostructure probe is successfully used to detect Fe^3+^ in living HeLa cells, which shows its great potential in bioimaging detection.

## Additional file

Additional file 1: Figure S1. a Magnetization curve of BHN-Fe_3_O_4_@SiO_2_. **b** UV-Vis spectra of BHN-IPTES, Fe_3_O_4_@SiO_2_, and BHN-Fe_3_O_4_@SiO_2_. (PDF 57 kb)

## Abbreviations

BHN: *N*-butyl-4-bis(2-hydroxyethyl) amino-1,8-naphthalimide
EDTA: Ethylenediaminetetraacetic acid
FT-IR: Fourier transform infrared
IPTES: 3-Isocyanatopropyl-triethoxysilane
TEM: Transmission electron microscopy
TEOS: Tetraethyl orthosilicate
TGA: Thermal gravimetric analysis
THF: Tetrahydrofuran
XRD: X-ray powder diffraction
