# Supplementary figures and images for: A Resumable Fluorescent Probe BHN-Fe3O4@SiO2 Hybrid Nanostructure for Fe3+ and its Application in Bioimaging
Source: Nanoscale Res Lett. 2017 Dec 19;12:629. doi: 10.1186/s11671-017-2392-2 (PMC5736514; doi:10.1186/s11671-017-2392-2)

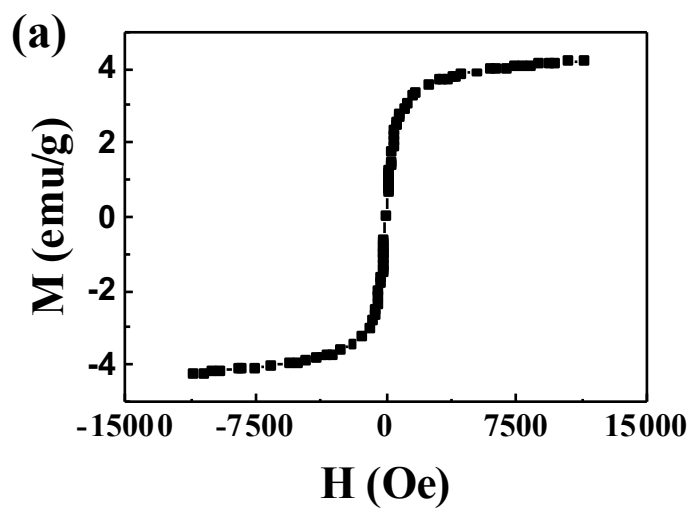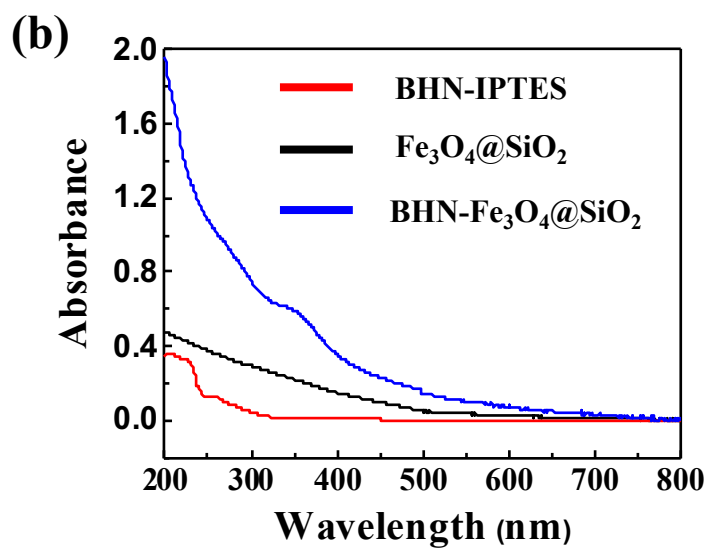

Supplement: Additional file 1: Figure S1. a — Magnetization curve of BHN-Fe3O4@SiO2. b UV-Vis spectra of BHN-IPTES, Fe3O4@SiO2, and BHN-Fe3O4@SiO2. (PDF 57 kb) [file 11671_2017_2392_MOESM1_ESM.pdf]
